# Efficacy of a Semi Automated Commercial Closed System for Autologous Leukocyte- and Platelet-Rich Plasma (l-prp) Production in Dogs: A Preliminary Study

**DOI:** 10.3390/ani10081342

**Published:** 2020-08-04

**Authors:** Roberta Perego, Eva Spada, Luciana Baggiani, Piera Anna Martino, Daniela Proverbio

**Affiliations:** 1 Veterinary Transfusion Research Laboratory (REVLab), Department of Veterinary Medicine (DIMEVET), University of Milan, via dell’Università 6, 26900 Lodi, Italy; luciana.baggiani@unimi.it (L.B.); daniela.proverbio@unimi.it (D.P.); 2 Department of Veterinary Medicine (DIMEVET), University of Milan, via dell’Università 6, 26900 Lodi, Italy; piera.martino@unimi.it

**Keywords:** platelet-rich plasma, closed system, dog, platelet, growth factors, activation

## Abstract

**Simple Summary:**

There are few publications on the subject of canine platelet concentrates and further studies are required to characterize these for clinical applications. The aim of this study determined platelet, erythrocyte, and leukocyte counts in canine leukocyte- and platelet rich plasma (L-PRP) produced using a commercial semi-automated closed system (CPUNT 20, Eltek group, Casale Monferrato, Alessandria, Italy). Whole blood (WB) from 30 healthy dogs was used. In 10 L-PRP bovine thrombin activated samples platelet-derived growth factor isoform BB (PDGF-BB) concentration was also measured. The CPUNT 20 produced clinically useful quantities of sterile canine L-PRP containing a high concentration of platelets (767,633 ± 291,001 μL, *p* < 0.001), with a 4.4 fold increase in platelet count, lower concentration of erythrocytes (528,600 ± 222,773 μL, *p* < 0.001) and similar concentration of leukocytes (8422 ± 6346 μL, *p* = 0.9918) compared with WB. Neutrophils, lymphocytes and monocytes average percent content in L-PRP was 14.8 ± 13.2, 71.7 ± 18.5 and 10.7 ± 6.4, respectively. Activated L-PRP has an average of 3442 ± 2061 pg/mL of PDGF-BB.

**Abstract:**

Background: To characterize the cellular composition (platelets, erythrocytes, and leukocytes) and determine platelet-derived growth factor isoform BB (PDGF-BB) concentration in canine leukocyte- and platelet rich plasma (L-PRP) produced using a commercial semi-automated closed system. Methods: Twenty milliliters of citrated whole blood were obtained from 30 healthy un-sedated canine blood donors and processed using a semi-automated completely closed commercial system (CPUNT 20, Eltek group, Casale Monferrato, Alessandria, Italy) according to the manufacturer’s instructions. Erythrocyte, leukocyte, and platelet counts were determined in both whole blood (WB) and resultant L-PRP. The PDGF-BB concentration was evaluated after bovine thrombin activation of 10 L-PRP samples. Results: This commercial system produced on average 2.3 ± 0.7 mL of L-PRP containing a high concentration of platelets (767,633 ± 291,001 μL, *p* < 0.001), with a 4.4 fold increase in platelet count, lower concentration of erythrocytes (528,600 ± 222,773 μL, *p* < 0.001) and similar concentration of leukocytes (8422 ± 6346 μL, *p* = 0.9918) compared with WB. L-PRP had an average of 3442 ± 2061 pg/mL of PDGF-BB after thrombin activation. Neutrophils, lymphocytes and monocytes average percent content in L-PRP was 14.8 ± 13.2, 71.7 ± 18.5 and 10.7 ± 6.4, respectively. Conclusion: Sterile canine L-PRP prepared using this semi-automated closed system is easy to obtain, produces a significant increase in platelet count compared to WB and contains a detectable concentration of PDGF-BB after activation. Additional in vitro and in vivo studies are needed to assess inflammatory markers concentration and the therapeutic efficacy of this L-PRP in dogs.

## 1. Introduction

Platelet-rich plasma (PRP) is a product derived from whole blood, characterized by platelet (PLT) concentrations higher than baseline in a small volume of plasma [1]. Growth factors such as platelet-derived growth factor (PDGF), transforming growth factor beta, vascular endothelial growth factor, basic fibroblastic growth factor, and epidermal growth factor usually contained in platelet α granules are released when platelets are activated or destroyed [2,3]. The release of these growth factors, that can act either individually or synergistically, into damaged tissue has the potential to facilitate cell proliferation, angiogenesis, tendon and wound healing, production of fibroblasts, collagen, osteoblasts, decreases the inflammatory reaction and accelerates the healing process [2,3]. The use of PRP for delivery of growth factors has emerged as a convenient method for promoting wound healing and tissue regeneration. The autologous nature of PRP preparations makes them safer and less expensive than allogenic cell-based regenerative therapies. As a result, there is widespread use of PRP therapy in human [4,5,6,7,8,9] and in veterinary medicine [10,11,12,13,14,15,16,17,18,19,20,21] prompting the need for development of simple and reproducible methods for preparation of high-quality PRP for clinical use.

In human medicine various authors have tried to define simple parameters for assessing the quality of platelet concentrates, a difficult target to achieve. Marx [22] has reported that the ideal PRP should have a platelet count with a 3- to 5-fold increase over whole blood or an ideal concentration of one million platelet/µL; similarly to Marx, Mazzucco [23] defines one million platelet/µL as a “reasonable compromise” for a quality platelet concentrate for non-transfusion use, while 300,000 platelets/µL is described by Anitua [24] as the minimum platelet concentration needed in a quality PRP. Platelet concentration is not the only important component of a PRP product; inclusion or exclusion of leukocytes not only defines a platelet concentrate [25,26] but has also been reported to affect the efficacy of the product and to have a significant effect on the inflammatory responses after clinical use of PRP [27,28,29,30,31,32].

The collection and processing of PRP should preserve the platelets in a quiescent state so that activation and subsequent growth factor release will be delayed until the moment of clinical use [33]. An important question is whether exogenous activation of PRP with thrombin or calcium should be performed before clinical application: the use of non-activated PRP would result in a greater and longer in time release of growth factors when the platelets are exposed to collagen and therefore activated in the injured site, but this is difficult to confirm in vivo and activation at the lesion site may not occur consistently [34,35,36]. The amount of growth factors available at the end of PRP production process depends on the techniques used (from blood collection to centrifugation) [37,38,39,40], on the biological variability of growth factor concentration among individuals [41,42] and on method of exogenous activation [36], but the relationship between platelet and growth factors concentration is far from clear [2,12,43].

Despite the popularity and advantages of PRP, there are conflicting reports in the literature regarding its clinical efficacy [44,45,46,47,48,49]. These differences may, in part, be attributable to variations in the composition of PRP preparations, therefore considerable research effort has been focused on optimizing human [28,38,50,51,52,53] and equine [54,55,56,57] protocols with regard to reproducible recovery, yield and cellular composition. However, there have been relatively fewer studies addressing optimization of PRP protocols in dogs. Many different manual or semi-automated methods for PRP production in dogs are described [58,59,60,61] achieving wide ranges of PLT concentration, but many of these studies have been performed on a small number of subjects [58,59,60] and few studies provide information about concentrations of platelet-derived growth factor or *p*-selectin externalization when PRP is activated [43,60,62,63,64,65]. Analysis of the previous studies indicates that it cannot be assumed that a system will produce the same PRP from canine blood as that obtained from human blood. Furthermore, the data highlight that the PRPs obtained by use of various commercially available systems can potentially differ in their characteristics [63]. Characterization of PRP products with specific focus on canine patients is needed to understand results of past, current, and future clinical applications or in vivo experiments [59] and more detailed evaluations including the reproducibility in cell content, growth factor concentration, and platelet activation status in the PRP have yet to be performed [63].

The aim of this study was to characterize the cellular composition (platelets, erythrocytes, and leukocytes) and determine the platelet-derived growth factor-BB (PDGF-BB) concentration after activation in the final product from a commercial semi-automated platelet-rich plasma closed system using canine blood.

## 2. Materials and Methods

### 2.1. Subjects

After approval by the Ethics Committee of the University of Milan (protocol number 13-01-15) and with owner-informed consent, citrated blood samples were collected from the cephalic vein of 30 healthy un-sedated adult canine blood donors (PLT count within the reference range), 17 males and 13 females, weighing between 20 and 45 kg and between 2 and 8 years of age (mean ± standard deviation: 4.2 ± 1.9 years). A variety of breeds were represented (e.g., Labrador retriever, Rhodesian ridgeback, Corso dog) and all dogs were admitted to the Veterinary Transfusion Research Laboratory (REVLab) of the Department of Veterinary Medicine for routine blood donor check-ups. All dogs were fasted for 12 h before blood sample collection.

### 2.2. Samples Collection and PRP Production

**Figure 1:**
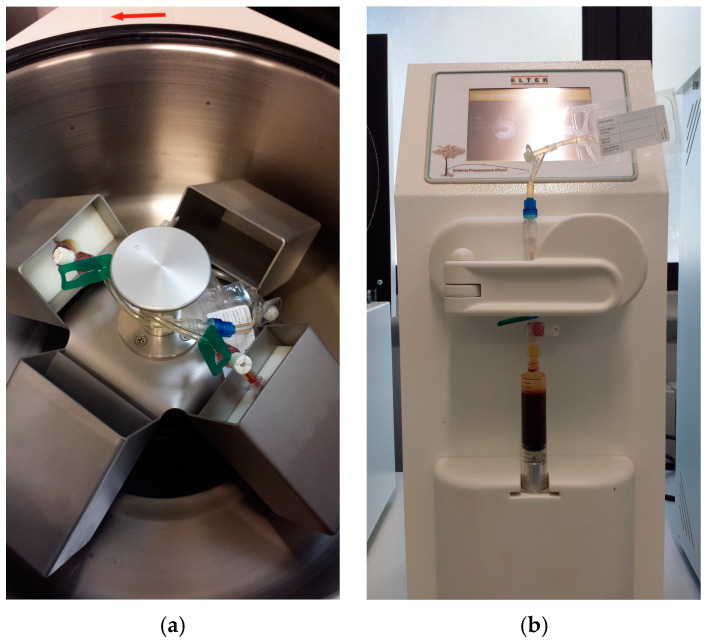
The dedicated centrifuge (**a**) and the automatic instrument for the separation of the PRP (**b**).

A closed semi-automatic platelet-rich plasma (PRP) collection and producing system for veterinary use (CPUNT 20, Eltek group, Casale Monferrato, Alessandria, Italy) was used, according to the manufacturer’s instructions. Briefly, the system consists of a sterile, single use, disposable collection kit for blood sampling, a dedicated centrifuge and an automatic instrument for the separation of the PRP (Figure 1).

**Figure 2:**
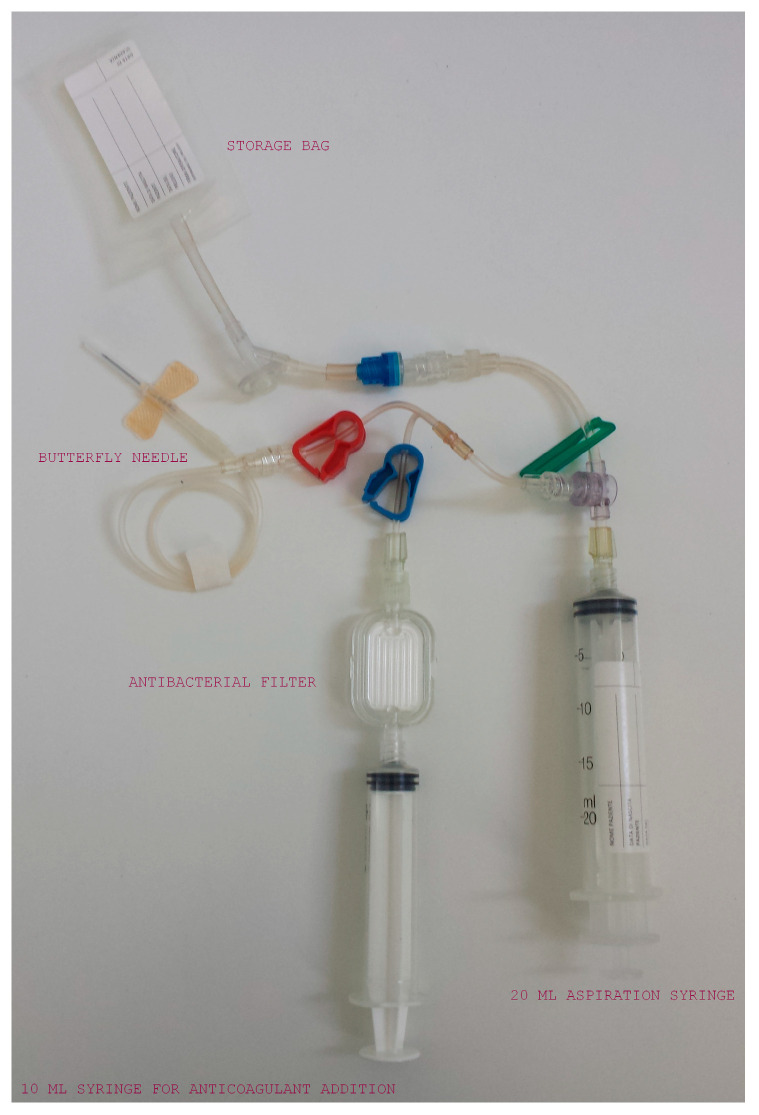
The collection kit: a butterfly needle connected to a 20 mL syringe with a port for addition of anticoagulant and an antibacterial filter (with attached 10 mL syringe) and a 10 mL bag for the storage of the PRP.

The collection kit comprises a butterfly needle (19G) connected to a 20 mL syringe for blood aspiration, with a port for anticoagulant addition and an antibacterial filter to preserve kit sterility, and a 10 mL bag for the storage of the PRP (Figure 2). All three connections are equipped with plastic clips that can be opened or closed as needed during the collection process. Two 10 mL syringes are also supplied in the sterile package together with the collection kit. Before starting the collection procedure, the three plastic clips (red, blue and green) must be closed, being careful to position the clip of the PRP storage bag close to the bag itself.

To prevent blood clotting, before blood sampling the plastic clip of the anticoagulant port was opened, 3 mL of 3.8% sodium citrate (not provided by the manufacturer) was inserted with the provided 10 mL sterile luer-lock syringe through the antibacterial filter and transferred to the aspiration syringe. The connection between the 2 tubes was then temporarily clipped.

The plastic clip of the blood aspiration port was opened and whole blood (WB) was collected from the cephalic vein of each subject up to the 20 mL mark on the aspiration syringe. The collection route was then clipped, the anticoagulant port was opened and another 1 mL of 3.8% sodium citrate was inserted.

**Figure 3:**
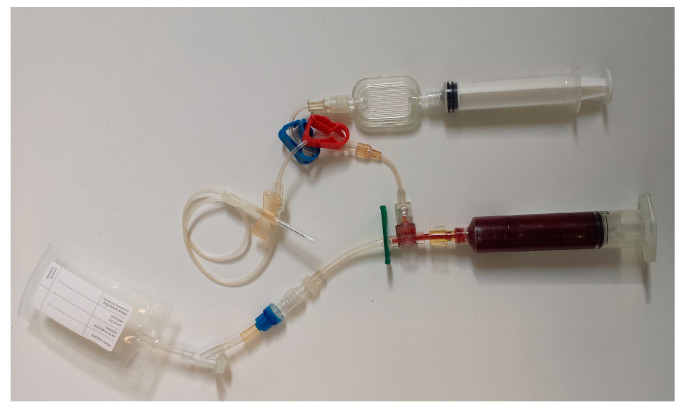
The part of the kit with the butterfly and the anticoagulant port (above) and the part of the kit dedicated to the production of the PRP (below).

After sample collection, the part of the kit with the butterfly and the anticoagulant port and a portion of the aspiration syringe piston were removed retaining only the part of the kit dedicated to the production of the PRP, i.e., the aspiration syringe connected to the 10 mL storage bag (Figure 3). These were centrifuged at 1200× *g* for 15 min, using the dedicated centrifuge equipped with special adapters. Immediately before centrifugation, the aspiration syringe was temporarily disconnected from the storage bag to withdraw 500 microliters of citrate WB for subsequent laboratory analysis. This step was performed only for the purposes of the study.

**Figure 4:**
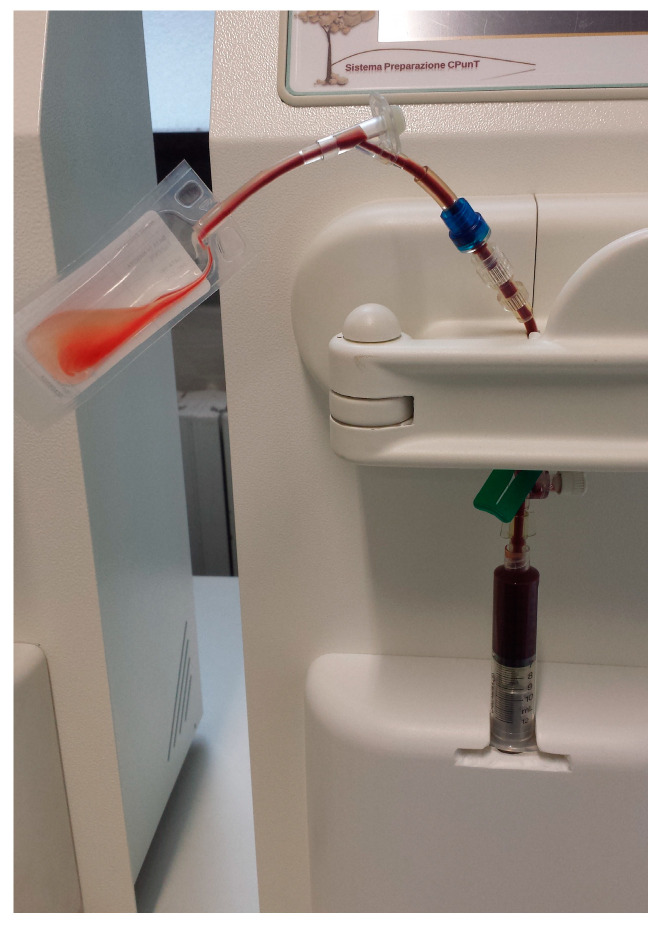
The supernatant plasma, the buffy coat and the surface of the erythrocytes layer pulled into the storage bag during the separation.

At the end of the centrifugation the syringe, in which erythrocytes, buffy coat and supernatant plasma layers are clearly visible, was gently removed from the adapter and the separating plastic clip between syringe and storage bag was opened. At this point, the kit was positioned in the automatic separation instrument. The movement of a plunger positioned under the syringe piston of the syringe under control of an optical reader isolated the supernatant plasma, the buffy coat and the surface of the erythrocytes layer into the storage bag (Figure 4). The aspiration syringe, now containing only the erythrocyte layer, was then separated from the bag.

**Figure 5:**
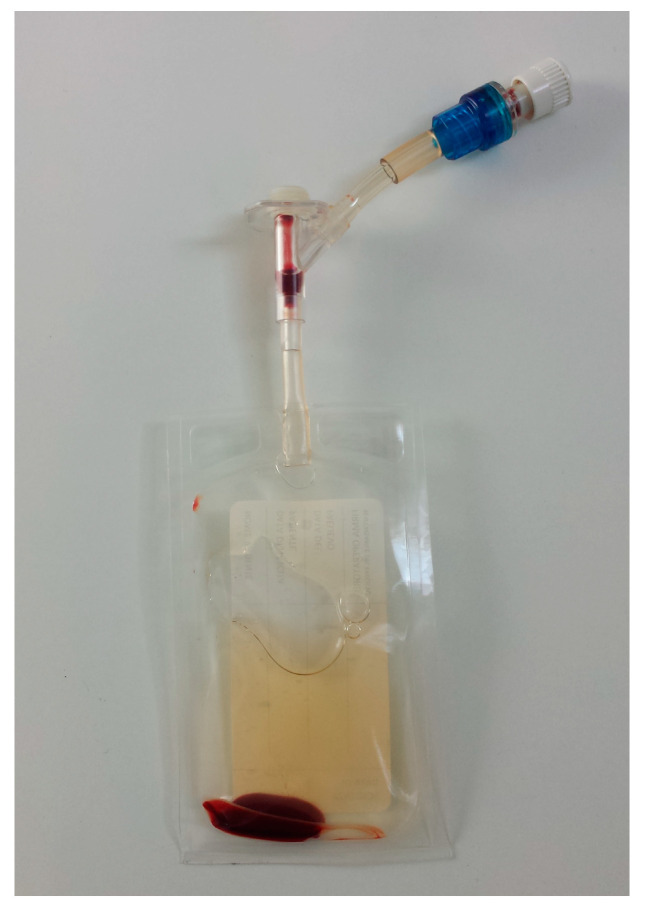
The pellet suspended in the platelet poor plasma (PPP) inside the storage bag.

The storage bag was then centrifuged again at × *g* for 5 min, using the appropriate slots of the same dedicated centrifuge. At the end of this second centrifugation, a pellet suspended in the platelet poor plasma (PPP) was formed inside the storage bag (Figure 5).

Finally, 75% of the supernatant PPP was removed using the provided 10 mL syringe through the appropriate perforable membrane, and the pellet was resuspended in 25% of the remaining PPP by gentle manual mixing.

**Figure 6:**
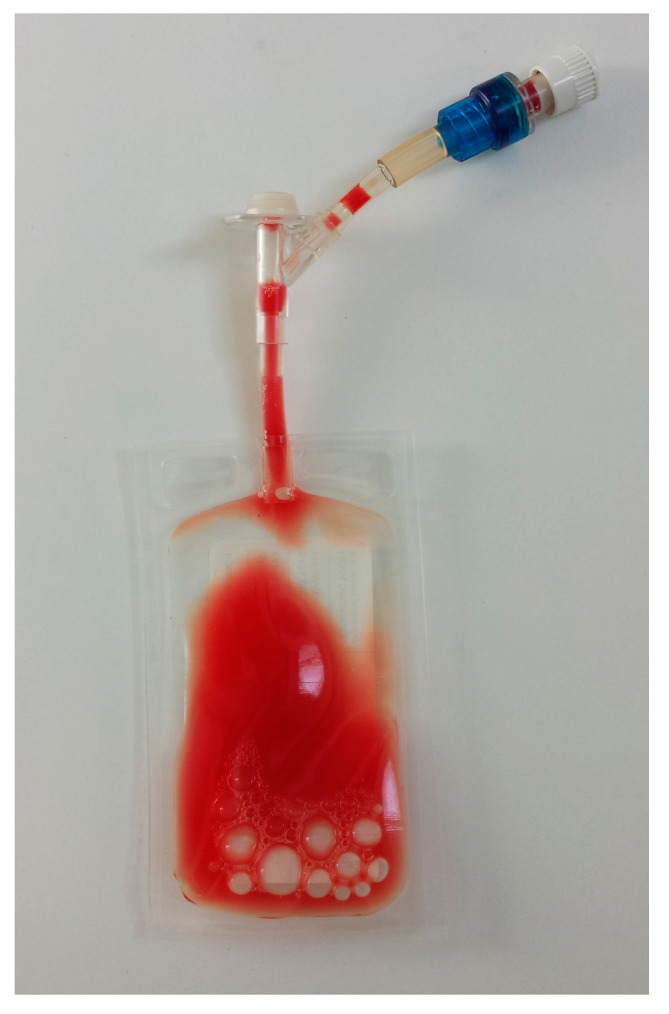
The final L-PRP inside the storage bag.

The leukocyte- and platelet-rich plasma (L-PRP) thus obtained (Figure 6) was collected through the latex perforable membrane with a sterile syringe and transferred to an empty tube for subsequent laboratory analyses.

### 2.3. Platelet and Leukocyte Counts

For each test subject (30/30), platelet count (PLT/μL) leucocyte count (WBC/μL) and erythrocyte count (RBC/μL), were calculated on WB and L-PRP by an automatic analyzer using optical and volumetric impedance measurements (Cell-Dyn 3500 analyzer, Abbott Diagnostics Europe), while neutrophils (cells/μL), monocytes (cells/μL) and lymphocytes (cells/μL) counts were calculated with the same instrument and checked with a manual differential count of blood smears in only 19/30 subjects, for technical reasons. In the same 19 subjects the smears were also used for assessment of possible platelet clumping. All sample were stored at room temperature on a laboratory blood rocker for a minimum of 5 min before counts were performed.

### 2.4. Microbiological Evaluation

This study was performed using a closed system designed to guarantee the sterility of the finished product. However, having temporally disconnected the aspiration syringe from the storage bag to withdraw 500 microliters of citrated WB for subsequent laboratory analysis (step not required by the method) and still wanting to test the validity of the method for further possible clinical uses, we performed bacteriological tests on seven, randomly chosen, L-PRP samples. One hundred microliters of L-PRP was plated by streaking onto blood agar plates (Microbiol, Cagliari, Italy) both for aerobic and anaerobic bacteria and then incubated at 35 ± 2 °C for at least 48 h [66].

### 2.5. Platelet Derived Growth Factor Evaluation

The PDGF-BB levels in 10 L-PRP samples were assessed after L-PRP activation of samples with. bovine thrombin. To each 1 mL of L-PRP, 50 μL of a bovine thrombin solution (BioPharm Laboratories LLC, Bluffdale, UT, USA) containing 500 IU/mL was added. After activation, the samples were kept at 37 °C in an incubator. The supernatant (after spontaneous clot retraction) of each activated L-PRP was collected at 3 h after activation and the concentration of PDGF-BB (pg/mL) was determined using a human ELISA kit (Human PDGF-BB Duoset DY220E, R&D Systems, Minneapolis, MN, USA) previously validated in dog [67] and used in others veterinary studies [41,68]. The mean detection sensitivity was <15 pg/mL. Measurements of the concentrations of PDGF-BB were performed in duplicate at 450 nm according to the manufacturer’s instructions.

### 2.6. Statistical Analysis

The normal distribution of parametric data was calculated using the D’Agostino-Pearson test and only RBC and lymphocytes values were found to be normally distributed. Results are presented as mean ± standard deviation. The statistical differences between mean values of PLT, WBC, neutrophils, lymphocytes, monocytes and RBC on WB and L-PRP were compared using Wilcoxon rank sum test or paired *t-*test depending on data distribution. The statistical differences between mean male and female PLT values were compared using Mann-Whitney test. The increase in platelet concentration in L-PRP over whole blood baseline values was calculated using the following equation: platelet count L-PRP/platelet count WB. Spearman’s coefficient of rank correlation (rho) was used to evaluate the relationship between cellular counts (PLT, WBC, neutrophils, lymphocytes, monocytes and RBC) in WB and in L-PRP. Correlation between cellular (PLT, WBC, neutrophils, lymphocytes, monocytes and RBC) and PDGF-BB concentrations in L-PRP were determined using Pearson test. For all tests significance was set at *p* < 0.05. Statistical analyses were performed using commercial software (MedCalc Software v.11.5.1 Mariakerke, Belgium).

## 3. Results

The mean volume of L-PRP obtained was 2.3 ± 0.7 mL. The average PLT, neutrophils, lymphocytes, monocytes and RBC values comparing WB and L-PRP were significantly different (Table 1). There was a 4.4-fold increase in the mean platelet concentration in L-PRP compared to baseline concentrations in WB: the highest value of PLT concentration was 1,898,000/μL (9-fold increase), the lower value was 304,000/μL (2-fold increased). There was no difference in L-PRP PLT concentration between males and females (*p* = 0.44). The mean PDGF-BB concentration in L-PRP samples after thrombin activation was 3442 ± 2061 pg/mL. Pearson correlation coefficient between cellular (PLT, WBC, neutrophils, lymphocytes, monocytes and RBC) and PDGF concentrations in L-PRP were 0.62 (*p* = 0.05), 0.60 (*p* = 0.06), 0.43 (*p* = 0.22), 0.50 (*p* = 0.14), 0.88 (*p* = 0.0008) and 0.23 (*p* = 0.52), respectively. All samples of L-PRP tested for bacteriological analysis were sterile.

## 4. Discussion

In this study canine L-PRP was prepared using a commercial semi-automated closed system. All the steps of the production method were easy to perform and the volume of platelet concentrate produced was more than sufficient (on average > 2 mL per subject were obtained) for therapeutic use: PRP has been used in quantities ranging from 0.6 to 3 mL in a variety of canine pathologies [69,70,71,72,73,74].

The fact that the system is closed means the final product is free from bacterial contamination, as confirmed by our microbiological evaluation This cannot be guaranteed in other double centrifugation methods with the exception of a laminar flow chamber under sterile conditions and with trained personnel. This is particularly important when canine platelet concentrates will be used therapeutically [58,60]. The proven sterility makes CPUNT system ideal for using the platelet concentrate for therapeutic regenerative purposes both in regions, such as joints or bone, that require a perfect asepsis and in regions, such as the oral cavity or skin, where asepsis is less critical.

Interestingly, it is also possible to withdraw an aliquot of the L-PRP obtained from the collection bag in a sterile manner, so as to be able to keep the remainder for dermatological or oculistic periodic therapeutic treatments in the same subject. Shelf life of the remaining product may be prolonged by freezing, as carried is out in human medicine [75,76], though studies demonstrate that freezing is detrimental to platelet morphology and function and continuous synthesis of growth factors [36]. In veterinary medicine, there are few studies considering extension of the shelf life by freezing of platelet concentrates [54,77,78,79,80] and in the dog there are no literature reports that confirm the best preservation method. In fact, there is only one research study focused on growth factors evaluation in which a small number of canine PRP samples was frozen at −80 °C and subsequently analyzed, demonstrating an increase in the growth factors following freezing similar to that obtained with calcium chloride activation [36] and one clinical study reporting PRP preservation (at −76 °C) before application to skin wounds in the dog, with a subsequent excellent clinical response [15].

The system utilized demonstrated a consistent ability to enrich platelets in autologous plasma (about fourfold) producing a significantly higher platelet count in L-PRP than in WB (*p* < 0.0001). Traditionally, a three- to five-fold increase in platelets has been considered an appropriate concentration for medical applications [22,51,57]. In this study, however, in 7 out of 30 samples the three times target platelet increase was not reached, with increases in platelet numbers of between 2- and 2.8-fold. No correlation was found between sex, age of the subjects, number of platelets in WB and L-PRP samples that did not reach the platelet target concentration. It is not uncommon in studies focused on preparation of PRP in dog, for not all samples to reach the predetermined enrichment value, with great variability between individual samples or very large reported standard deviations [13,58,81], as happened also in our study in which the standard deviation for platelet concentration was large. In a study on the assessment of a PRP produced with a commercial centrifugation and platelet recovery kit the minimum platelet enrichment was still considered to be within an appropriate range for medical application with 90% of samples falling within the range of 4.7- to 8.1-fold enrichment [63]. According to previous literature [24], PRP products must achieve a platelet count of at least 300 × 10^3^ platelets/μL to be in the therapeutically effective range and this was reached by the seven sampled in this study that failed to concentrate 4-fold. This proposed minimum platelet number for PRP has been extrapolated from the human literature and key species differences in average platelet counts and platelet physiology preclude an accurate evaluation of literature data [82]. The ideal enrichment of platelets in veterinary PRP remains unknown and may depend on the species, the specific disease and the site of application for the regenerative therapy. In man it has been shown that too many platelets may be detrimental to tissue repair [25,30,31,57,83,84,85], but to date no studies have been conducted to investigate this in dogs [59].

There was no statistical difference in L-PRP PLT concentration between the two sexes, but, as already found in human patients and horses [41,42] as well as the dog the PLT count in P-PRP was on average higher in females than males.

In our study, the L-PRP system utilized significantly decreased the RBC concentration compared with WB (*p* < 0.001). Reducing RBC concentration is thought to be important when developing the ideal PRP product [86]. A recent study revealed that an increased RBC concentration in PRP increases the concentrations of unwanted inflammatory mediators, specifically IL-1 and TGF-α. This study also showed that when synoviocytes are treated with RBC concentrate there were significantly more synoviocyte deaths when compared with leukocyte-rich PRP, leukocyte-poor PRP, and phosphate-buffered saline [86].

The CPUNT 20 separation instrument has two modes of use: the production mode of the PRP with the inclusion of leukocytes, which allows L-PRP (leukocyte- and platelet-rich plasma) collection and the production mode without the inclusion of leukocytes, which results in production of P-PRP (pure platelet-rich plasma), according to Dohan Ehrenfest’s classification. In this study we used the PRP production modality with the inclusion of leukocytes, obtaining an L-PRP with an average WBC not statistically different from that in WB, with a moderate positive correlation between WBC concentration in WB and L -PRP (rho = 0.387, *p* = 0.03). The average WBC in our L-PRP did not reach the limit of twice baseline value defined by some authors [20,58] although the high standard deviation underlines a great variability between the samples. Leukocyte concentrations may influence the effects of PRP therapy, but their role is still a matter of debate. Leukocytes- and platelet-rich plasma has been associated with increased pro-inflammatory mediators and a catabolic effect [29,87,88,89]. P-PRP has been thought to be more beneficial than L-PRP in maintaining tendon homeostasis and counteracting inflammation associated with osteoarthritis [27,31,86,89]. Interestingly, however, a human study found both groups improved with no differences in pain or functional scores between P-PRP and L-PRP for intra-articular knee injections [90]. Other authors have instead shown how the presence of leukocytes in an injectable preparation of PRP can be useful for increasing the in situ production of growth factors [28,91], for the potential analgesic effect through the release of different chemokines, anti-inflammatory cytokines (IL-4, IL-10 and IL-13) and opioid peptides (β-endorphins, encephalins and dinorphine-A), to promote the inhibition of pain in a clinically relevant way [26,92] and to increase the important antimicrobial role in PRP [93,94].

In our study, in L-PRP we achieved a decrease in neutrophils and an increase in lymphocytes and monocytes statistically significant compared to WB, with a positive correlation between lymphocytes concentration in WB and L-PRP (rho = 0.558, *p* = 0.016). The potentially deleterious effects of leukocytes are largely attributed to the presence of neutrophils [25,27,29,31], while the effect of monocytes and lymphocytes remains largely unknown. Further investigation is needed regarding various leukocyte lineages, their concentrations, and their effects on PRP utilization evaluated according to the purpose of use (e.g., regenerative, antimicrobial, analgesic). Based on the sparse canine literature available it is already apparent that leukocyte profiles differ depending on the PRP processing method; however further comparison of the current canine literature remains difficult until a standardized methodology for assessing cellular enrichment or growth factor concentration is adopted for kit analysis [63]. High WBC counts are generally accepted in platelet concentrates used for autologous topical application [58,87,95]. Thus, the desirable WBC count is still a matter of speculation [25] and to date there are no studies that clarify the significance of leukocytes in canine PRP and the optimal ratio of platelet to leukocytes. Finally, the integrity of leukocytes seems to be of considerable importance: centrifugation can activate or destroy white blood cells and/or stimulate the inflammatory state [26].

In our samples there was no any correlation in PLT count between WB and L-PRP when the specific values for each L-PRP and WB samples were analyzed in line with previous studies in man [96], but in contrast to what has been reported in the horse [54]. This conflicting result could depend on the PRP production process used and/or on the different species evaluated and other studies are needed in the dog to understand the validity of this lack of correlation.

The main objective for delivery of platelets is to increase growth factor concentrations to the affected tissue; however platelet enrichment does not necessarily result in increased delivery of growth factors since degranulation can occur during the centrifugation, leading to a plateau in growth factors that depend on platelet activation [97]. The amount of growth factors available at the end of PRP production process therefore depends on the particular technique used to obtain the PRP [37,38,54,98,99]. However, the platelet growth factor concentrations in PRP could be influenced by other methodological aspects, such as the type of anticoagulant used during blood collection, the ratio of blood to anticoagulant, the type of activating agent and the activation protocol [32,34,36,43,53,68]. Among the numerous growth factors present in platelet α granules, we selected PDGF-BB as an index of activated platelets because it is only secreted by activated platelets and is not found in plasma. Thus, its concentration should represent platelets activation and alpha granules release [60]. In our study the PDGF concentration in activated L-PRP is similar to that found in previous studies in dog [60,65], demonstrating a correct release of growth factors following platelet activation with bovine thrombin. The relevance of this to clinical application remains tenuous as in vivo activation depends on the site of injection, and bovine thrombin cannot be used in vivo due to the potential for adverse immunological reactions seen in human medicine [22,100]. Unfortunately, in our study the PDGF-BB dosage was measured in only 10 samples and this may have affected our results on correlations with sex or breed, which have previously been studied in man, horses and rabbits, with different results [41,96,101].

This study shows a positive and significative correlation between PDGF-BB concentration and PLT concentration in L-PRP (Pearson index 0.62 with *p* = 0.005). The relationship between platelet and PDGF concentrations is far from clear. Some authors report correlation between platelet and PDGF concentrations [43,60] and others do not [12,37,42,64]. Franklin et al. [36] suggest that activation had a greater effect on growth factor concentration than did cellular composition. Several factors might contribute to these discordant results, for example: manipulation—induced platelet stress and variable susceptibility of platelets to stress. Furthermore the biological variability of growth factor and platelet concentration in PRP among individuals must be taken into account [41,42,96,102]. The presence of a positive correlation between WBC and PDGF-BB and monocytes and PDGF in L-PRP (Pearson index 0.6 with *p* = 0.06 and 0.88 with *p* = 0.0008, respectively) in our results seems to corroborate the theory that the quantity of white blood cells in the PRP also influences the concentration of growth factors [32,63,91].

A limitation of our study was that we did not measure PDGF-BB concentrations in whole blood. We could speculate that the concentration of PDGF-BB might increase in L- PRP, after activation, in our samples by comparing our values with those found in WB in the literature [65], but we cannot be sure, also considering that the blood sampling site in the dog seems to affect the activation status of the platelets. [63] Further studies will be needed to investigate this. Another limitation was that we did not measure whether platelets were fully activated in the L-PRP, perhaps with microscopical morphological evaluation of blood smears or with *p*-selectin dosage [63,64]. Among the PRPs with the same number of platelets, the concentrations of growth factors will depend on the number of activated platelets [60] and there is a correlation between activated platelets and quantitative growth factors, as reported in previous studies in canine PRP obtained with other methods [64]. To activate as many platelets as possible, various agonists (collagen, ADP and thrombin, etc.) and the ratio between PRP and agonist should be further studied in dogs, as suggested by previous authors [60].

Another limitation of our study was that we did not evaluate some key inflammatory markers, such as interleukin—1β or TNF-α, and their correlation with WBC and PLT content in L-PRP. The concentration of these catabolic cytokines, that seems differ in various PRPs, may be clinically relevant. A final limitation was that we didn’t evaluate the production mode without the inclusion of leukocytes of the CPUNT 20 separation instrument, which results in production of P-PRP, according to the manufacturer’s instructions; it would have been interesting to compare the characteristics of the two options provided by the kit with canine samples.

## 5. Conclusions

The closed semi-automated system utilized in this study produced a fourfold mean increase in platelets, but with a large standard deviation and reduced the relative percentage recovery of erythrocytes, with a similar concentration of leukocytes but with a lower concentration of neutrophils as in baseline WB. The autologous L-PRP obtained had PDGF-BB concentration comparable to previous data in the literature after activation with bovine thrombin, was sterile and was produced in a sufficient volume to allow clinical use in various fields of canine medicine. Further studies are recommended to evaluate inflammatory markers and clinical application of this L-PRP product in dogs.

## Figures and Tables

**Table 1:** Mean and standard deviation of whole blood (WB) and leukocyte-platelet rich plasma (L-PRP) cell counts.

Cellular Type (Number of Subject)	WB (%)	L-PRP (%)	*p* (° Wilcoxon; * *t*-Test)	Spearman’s Coefficient
Platelet (μL) (30/30)	185,300 ± 39,576	767,633 ± 291,001	<0.0001 °	rho: 0.259 *p*: 0.1673
Leucocytes (μL) (30/30)	8167 ± 3425	8422 ± 6346	0.9918 °	rho: 0.387 *p*: 0.034
Neutrophils (μL) (19/30)	6303 ± 3459 (63.7)	1250 ± 1960 (14.8)	<0.0001 °	rho: 0.428 *p*: 0.0762
Lymphocytes (μL) (19/30)	2500 ± 1329 (29.3)	5231 ± 2620 (71.7)	0.0001 *	rho: 0.558 *p*: 0.016
Monocytes (μL) (19/30)	248 ± 174 (3)	823 ± 792 (10.7)	0.0008 °	rho: −0.164 *p*: 0.52
Erythrocytes (μL) (30/30)	5,907,433 ± 704,099	528,600 ± 222,773	<0.0001 *	rho: −0.108 *p*: 0.57

Legend: mean values of PLT, WBC, neutrophils, lymphocytes, monocytes and RBC on WB and L-PRP were compared using Wilcoxon rank sum test (°) or paired *t*-test (*) depending on data distribution. Spearman’s coefficient of rank correlation (rho) was used to evaluate the relationship between platelet, leucocytes, neutrophils, lymphocytes, monocytes and erythrocytes counts in WB and L-PRP.
